# How pregnancy can affect autoimmune diseases progression?

**DOI:** 10.1186/s12948-016-0048-x

**Published:** 2016-09-15

**Authors:** Marie-Pierre Piccinni, Letizia Lombardelli, Federica Logiodice, Ornela Kullolli, Paola Parronchi, Sergio Romagnani

**Affiliations:** 1 Center of Excellence for Research, Transfer and High Education DENOTHE of the University of Florence, Florence, Italy; 2 Department of Experimental and Clinical Medicine, University of Florence, Largo Brambilla 3, 50134 Florence, Italy

**Keywords:** Autoimmunity, T helper cells, Th1, Th2, Th17, Th22, Tfh pregnancy, Abortion, Allograft

## Abstract

Autoimmune disorders are characterized by tissue damage, caused by self-reactivity of different effectors mechanisms of the immune system, namely antibodies and T cells. Their occurrence may be associated with genetic and/or environmental predisposition and to some extent, have implications for fertility and obstetrics. The relationship between autoimmunity and reproduction is bidirectional. This review only addresses the impact of pregnancy on autoimmune diseases and not the influence of autoimmunity on pregnancy development. Th17/Th1-type cells are aggressive and pathogenic in many autoimmune disorders and inflammatory diseases. The immunology of pregnancy underlies the role of Th2-type cytokines to maintain the tolerance of the mother towards the fetal semi-allograft. Non-specific factors, including hormonal changes, favor a switch to Th2-type cytokine profile. In pregnancy Th2, Th17/Th2 and Treg cells accumulate in the decidua but may also be present in the mother’s circulation and can regulate autoimmune responses influencing the progression of autoimmune diseases.

## Background

Autoimmune diseases include approximately 80 different disorders. Although, individually, each autoimmune disease affects a small number of individuals, as a whole, it is estimated that its prevalence is between 7.6 and 9.4 % [1]. It is well accepted that a disease can be classified as autoimmune if one shows that an immune response to a self-antigen causes the disease pathology. Indeed, autoimmune disorders are characterized by tissue damage, caused by self-reactivity of different effectors mechanisms of the immune system, namely antibodies and T cells. Their occurrence may be associated with genetic and/or environmental predisposition [2, 3] and to some extent, have implications for fertility and obstetrics.

The relationship between autoimmunity and reproduction seems to be bidirectional. Accordingly, autoimmune diseases may selectively affect women in their reproductive years and conversely pregnancy may affect the expression of autoimmune diseases. Thus, autoimmunity may have an influence on pregnancy outcomes. As the matter of fact, Gleicher et al. [4], performed PubMed, Google Scholar and Medline searches for the years 2000–2010 under various key words and phrases, referring to effects of autoimmunity/autoimmune diseases on pregnancy/pregnancy outcomes/pregnancy rates/reproduction/reproductive outcomes/fertility/infertility/fertility treatments/infertility treatments, and similar terms, toward significant impacts of autoimmunity on female reproductive success. They reported that autoimmunity not only increases miscarriage risks but also reduces female fecundity and infertility treatment success. However, pregnancy may have an influence on autoimmune diseases improvement or worsening. During pregnancy many autoimmune diseases go into remission, only to flare again in the early post-partum period. For example, Graves disease is an autoimmune thyroid disease which ameliorates during pregnancy, only to relapse post partum.

This review only addresses the impact of pregnancy on autoimmune diseases and not the influence of autoimmunity on pregnancy development.

## Pregnancy is related to T cell mediated-responses towards conceptus

The fact that women can successfully carry a conceptus, which is liken to an allograft, to full term without rejection is one of the most remarkable aspects of pregnancy.

Although conceptus/trophoblast does not express HLA class II molecules, it exhibits HLA class I molecules, the polymorphic HLA-C molecules, together with the non-polymorphic HLA-G and HLA-E. For the presence of paternal class I HLA-C molecules on the fetal-derived trophoblast cells, that invade the maternal *decidua basalis*, the conceptus has been considered to be a semi-allograft. After presentation of paternal alloantigens by maternal antigen presenting cells (APCs), the maternal T cells specific for these alloantigens, [5] could proliferate and secrete cytokines, promoting the activation of allograft rejection or tolerance mechanisms, respectively responsible for pregnancy failure or fetal survival.

In fact, on the basis of the profile of cytokines produced, T helper cells are classified in T helper (Th)1, Th2 and Th17 cells [6, 7]. CD4+ Th1 cells produce interleukin (IL)-2, tumor necrosis factor (TNF)-β and interferon (IFN)-γ and are the main effectors of phagocyte-mediated host defense, which are highly protective against infections sustained by intracellular pathogens. On the other hand, CD4+ Th2 cells, which are mainly responsible for phagocyte-independent host defense against extracellular pathogens, including nematodes, produce IL-4 (which together with IL-10 inhibit several macrophage functions and together with IL-13 produced by Th2 cells, stimulates IgE antibody production) and IL-5 (which promotes growth, differentiation and activation of eosinophils) [6]. An additional subset of CD4+ T helper cells named Th17, which produce IL-17A, IL-17F, IL-21, IL-26 and IL-22 [7], is protective against extracellular bacteria and may also play a role in inflammation [8]. A part of human IL-17A-producing cells were found to also produce interferon (IFN)-γ (they are named Th17/Th1) and both Th17 and Th17/Th1 exhibit plasticity towards Th1 cells in response to IL-12 produced by APCs [8]. Another part of the Th17 cells could also produce IL-4 (they are named Th17/Th2) in response to IL-4 present in the microenvironment of these cells or HLA-G5 produced by trophoblast cells and embryo at feto maternal interface [9, 10].

These three types of effector T helper cells play a central role in acute allograft rejection and tolerance. IFN-γ and IL-17 has been detected in rejecting allografts [11–13], indicating that some Th1- and Th17-dependent effector mechanisms play a central role in acute allograft rejection of transplanted tissues [14]. The production of the Th2-type cytokine IL-4 and IL-10, appears to be central for the induction and the maintenance of allograft tolerance [11, 15]. These findings indicate that Th1-type and Th17-type cytokines, that promote allograft rejection, may compromise pregnancy, whereas the Th2-type cytokines inhibiting in particular the Th1 responses, promote allograft tolerance and therefore may improve pregnancy success. Moreover, T reg cells, which dampen all the T helper responses has also been reported to be responsible for fetal alloantigens tolerance [16] and could induce fetoallograft tolerance through the production of IL-10 and TGF-β [17]. However recent studies in mice suggest that T reg cells play an important role in response to semen independently of placental antigen exposure, in preparing endometrium for implantation dampening uterine inflammation [18].

In mice many evidence confirmed the role of Th1- and Th2-type cytokines present at fetomaternal interface on the development of pregnancy [19–22]. Studies performed on unexplained recurrent abortion (URA), which is characterized by the loss of three or more consecutive pregnancies in the first trimester of pregnancy (with normal karyotype of the conceptus) has been chosen as model to study the first trimester of human pregnancy. Though multiple causes of URA are known (including structural, hormonal, infectious, autoimmune and thrombophilic causes), after evaluation, roughly half of all cases remain unexplained. The URA cases remained unexplained shed a light on the potential role of decidual effector CD4+ T helper on the maintenance of pregnancy.

Indeed, we reported a defect of IL-4 production by both CD4+ and CD8+ T cell clones and a defect of IL-10 by CD4+ T cell clones generated from the decidua of women suffering from URA undergoing a spontaneous abortion in comparison with women with successful pregnancy [23]. Thus, we found in URA a defective production of Th2-type cytokines which are essential for the fetal alloantigen tolerance in successful pregnancy. More recently, we reported that the purified human decidual CD4+ T cells obtained from successful pregnancy, which have not been activated in vitro, produce spontaneously IL-4, whereas they do never produce IFN-γ spontaneously, but only after stimulation with immobilized anti-CD3 monoclonal antibodies [24]. We also demonstrated that the purified CD4+ T cells were previously activated by the trophoblast cells in situ and expressed without any additional in vitro stimulation high levels of activation markers (CD25, CD69 and HLA-DR) [24]. These results confirm the prevalence of Th2 cells activated by trophoblast antigens at the feto-maternal interface [23, 25, 26]. Progesterone, which, at concentrations comparable to those present at the feto-maternal interface during pregnancy, is a potent inducer of production of IL-4 [27] could be at least in part responsible for a Th2 switch at feto-maternal interface.

Excessive Th17 activity has also been described to promote spontaneous abortion. Accordingly, Th17 cells has been reported to increase in decidua of patients with URA compared to healthy pregnant women [28, 29]. However, this significant increase seems to occur only in inevitable abortion with genital bleeding [29] and was suggested to be the consequence rather than the cause of miscarriage.

Very recently, we reported that an associated production of IL-4 and IL-17 is exhibited by a large number of decidual CD4+ T cells (named Th17/Th2 cells) in successful pregnancy and at embryo implantation site whereas pathogenic decidual Th17/Th1 cells are prevalent in URA and far from the embryo implantation site [10]. The differentiation of Th17 cells into Th17/Th2 cells is due to soluble HLA-G5, the soluble isoform of HLA-G, a non polymorphic Class I molecule produced by embryo and trophoblast cells in normal human pregnancy [10]. Thus, it seems that Th17 cells do not show a pathogenic role for pregnancy, but a beneficial role, when they also produce IL-4 whereas Th17/Th1 are pathogenic for pregnancy. In the intrauterine environment (both fetal and amniotic compartments) infection-related immunity seems to be preferentially directed towards combating extracellular microbial pathogens (responsible for a five fold increase of fetal resorption) with Th17-mediated immune responses [30] suggesting that Th17/Th2 cells could be essential for the success of pregnancy, because they may promote an adequate response to protect the mother against dangerous extracellular pathogens and induce fetoallograft tolerance together with Th2 cells and T reg cells.

Thus, T cells functions exacerbated during pregnancy characterized by a tolerogenic Th2-type profile, could have an influence on autoimmune disease improvement or worsening.

## Autoimmunity is related to T cell mediated-responses

CD4+ T helper cells are critical for proper immune cell homeostasis and host defense, but are also major contributors to pathology of autoimmune and inflammatory diseases. Since the discovery of the Th1/Th2 dichotomy, many additional Th subsets were discovered, each with a unique cytokine profile, functional properties, and presumed role in autoimmune tissue pathology. This includes principally Th1, Th2, Th17 and Treg cells which are characterized by specific cytokine profiles described above. Cytokines, which are often categorized into pro-inflammatory and anti-inflammatory produced by these T helper subsets play a critical role in directing the effector response. The different T helper subsets in terms of cytokine profiles influence and shape the immune response, and could promote autoimmune and inflammatory diseases [31]. Therefore, autoimmune diseases develop as a result of abnormalities in immune response mediated by activated T cells-derived cytokines. Accordingly, IFN-γ produced by Th1 cells, has long been associated with pathology of several autoimmune diseases including autoimmune type 1 diabetes, multiple sclerosis (MS) and rheumatoid arthritis (RA). The association IFN-γ and autoimmune disorders is not surprising because IFN-γ is a potent proinflammatory cytokine which has a number of important roles including, increasing major histocompatibility gene complex (MHC) class I (MHC-I) and class II (MHC-II) antigen presentation, increasing the expression of toll-like receptors (TLR) by innate immune cells, promoting immunoglobulin G class switching, promoting the induction of chemokine secretion, macrophage activation and increased phagocytosis. Even before the discovery of Th1 cells, evidence for IFN-γ having detrimental effects on autoimmune diseases was provided by the observation that administration of IFN-γ to MS patients was deleterious and resulted in exacerbation of the disease [32].

However, there is some debate whether Th1 cells only play a pathogenic role in autoimmune diseases, or whether they also contribute to protective/anti-inflammatory immune responses. Experimental allergic encephalomyelitis (EAE) is an animal model of inflammatory demyelinating disease of the central nervous system. It is widely studied as an animal model of the human central nervous system demyelinating diseases, including MS. Experiments testing the impact of genetic deletion of IFN-γ on EAE showed that animals lacking IFN-γ developed disease with increased severity compared with IFN-γ sufficient controls [33]. Several hypotheses have been proposed to account for the apparent anti-inflammatory properties of IFN-γ including the downregulation of lymphocyte trafficking into the draining lymph-nodes [34], the control of T cell clonal expansion via induction of apoptosis [35] and the induction of indoleamine 2,3-dioxygenase (IDO), which exerts anti-inflammatory effects in lymph nodes and tissues [36].

Another T helper subset characterized by the production of IL-4, IL-5 and IL-13, the Th2 subset can promote pathology of several different autoimmune diseases, particularly those which are associated with humoral immune responses. Indeed, studies have demonstrated that aberrant and continued IL-4 expression in vivo can rescue autoreactive B cells from apoptosis, enhance their survival, and induce activation of autoreactive B cells and thereby promote autoimmune diseases [37]. Additionally, even in Th1 mediated immunopathology, Th2 cells may induce the generation of autoantibodies and enhance pathology. In contrast, Th2-type cytokines can mediate protection either by directly suppressing Th1/Th17 development via IL-4/IL-13 respectively, or by counteracting Th1-mediated inflammation. IL-4 is known to strongly suppress the development of Th1 cells even in an environment with high levels of IFN-γ, thereby antagonizing Th1 cell functions [38]. Anti-inflammatory properties of IL-4 include the inhibition of Th1-activated macrophages and suppression of the secretion of several potent pro-inflammatory mediators including IL-1, TNF and reactive oxygen species (ROS).

Even prior to the discovery of Th17 cells, which produce IL-17A, IL-17F and IL-22, IL-17 was noted to be overexpressed in a number of inflammatory/autoimmune conditions including MS [39], RA [40, 41], systemic lupus erythematosus (SLE) [42, 43] and airway inflammatory diseases [44] and thus has been implicated in their pathogenesis. Models of autoimmune diseases have substantiated Th17 cells as important contributors to tissue pathology and to the promotion of antibody responses. Much of the pathogenic functions of Th17 cells have been attributed to the secretion of IL-17, including the induction of the release of proinflammatory cytokines including TNF-α and IL-1β, the enhancing B cell functions, the recruitment of neutrophils and the activation of innate immune cells. Moreover, IL-17 induces the secretion of chemokines and other inflammatory mediators, the expression of adhesion molecule (ICAM-1), prostaglandin E2, as well as promoting tissue damage through the induction of matrix metalloproteinases and antimicrobial-peptides. In fact, increased IL-17 production sustains a pro-inflammatory environment and can cause excessive tissue damage [45]. Recently, it has been suggested that Th17 cells that have shifted towards Th1-phenotype seem to be more aggressive and more pathogenic than Th17 unshifted cells particularly in inflammatory diseases as MS, RA, psoriasis or inflammatory bowel diseases. Nonetheless, IL-17 could be dispensable for the development of organ-specific autoimmunity, as in EAE [46]. Although IL-17 could be dispensable, IL-23, which promotes the stability of Th17 cells, and T-bet, key transcription factor of Th1 cells, which regulates the expression of IL-23 receptor, are essential for autoimmune disorders.

Very recently, the newly identified T helper cell 22 (Th22) has been involved in autoimmune diseases pathogenesis. Th22 is a subset of CD4+ T helper cells obviously discrete from Th17 and Th1 subsets by production of interleukin (IL)-22 but not IL-17 or IFN-γ, and also with distinguished expression of aryl hydrocarbon receptor (AHR) as the key transcription factor. This T helper subset, by producing pro-inflammatory cytokines such as IL-22 and tumor necrosis factor-α (TNF-α), is implicated in the pathogenesis of autoimmune disease including RA, SLE, Behçet’s disease, type 1 diabetes, immune thrombocytopenia [47], psoriasis and MS [48]. Thereby, Th22 cells and in particular IL-22 are implicated as a potential therapeutic target in autoimmune diseases.

Finally, not only Th1, Th2, Th17 and Th22 cells have been implicated in the pathogenesis of autoimmune disorders, the new subset follicular helper T cells (Tfh) seems to have a role in the pathogenesis of different autoimmune disorders [49]. Tfh has many features in common with Th17 cells, such as the ability to produce IL-21 and to express the IL-23 receptor (IL23R), the inducible co-stimulatory molecule ICOS, and the transcription factor c-Maf, all of them essential for expansion and establishment of the final pool of both Th17 and Th22 subsets. Tfh cells differ from Th17 by their ability to home to B cell areas in secondary lymphoid tissue through interactions mediated by the chemokine receptor CXCR5 that they express, and its ligand CXCL13. These CXCR5+ CD4+ T cells are considered an effector T cell type specialized in B cell help. The role of Tfh cells and its primary product, IL-21, on B cell activation and differentiation is essential for humoral immunity against infectious agents. However, when deregulated, Tfh cells seem to contribute to exacerbated humoral response and autoantibody production in autoimmune diseases. Accordingly, the presence of circulating Tfh cells as a potential biomarker of disease has been studied in various autoimmune conditions, including myasthenia gravis (MG), autoimmune thyroiditis, Sjögren’s syndrome (SS), RA, MS, SLE, ulcerative colitis, Crohn’s disease, ankylosing spondylitis, type 1 diabetes mellitus, autoimmune hepatitis, primary biliary cirrhosis and juvenile dermatomyositis. Alteration of Tfh cells have been reported in patients with various autoimmune diseases, such as RA, SLE and autoimmune thyroid diseases, where Tfh cells are present at increased frequency and show positive correlation with serum autoantibody titer. Therefore, a better understanding of the biology and the roles of Tfh cells is expected to contribute in clarifying the role of this subset in the pathogenesis of autoimmune disorders [49].

Some cytokines produced by non-T cells could stimulate the inappropriate activities of cells implicated in the pathogenesis of autoimmune diseases. Interleukin-27 (IL-27) is a member of the IL-12 family, is produced by activated antigen-presenting cells and plays an important role in the regulation of CD4+ T cell differentiation and immune response. IL-27 activates multiple signaling cascades, including the JAK-STAT and p38 MAPK pathways. Several studies have revealed that IL-27 promotes the differentiation of Th1 and Tr1, but inhibits Th2, Th17, and Treg cells. IL-27 displays both pro- and anti-inflammatory activities in different autoimmune diseases. The pro-/anti-inflammatory activity of IL-27 is influenced by the underlying immune effector pathways, the phase of the disease, the presence or absence of counter-regulatory cytokines/T cell subsets, and the tissue/cell type under study. Meka et al. [50] discussed the role of IL-27 in RA, MS, colitis, SLE, psoriasis, type 1 diabetes, and uveitis. Most of this information is derived from experimental models of these autoimmune diseases.

Despite a spectrum of outcomes in various autoimmune diseases, mostly anti-inflammatory and immunomodulatory effects of T cell cytokines have been observed in these disorders. Accordingly, T cell cytokines, but also inducers/inhibitors of some T cell-secreted cytokines represent a novel, promising target/agent for the treatment of autoimmune diseases.

## T cell responses in autoimmune disorders are influenced by pregnancy

In the light of what described before, Th1 and Th17-type autoimmune disorders could be improved when there is a rise in Th2-type cytokines in pregnancy (e.g. RA, Graves disease and MS [51–54]) and Th2-type autoimmune disorders could get worse in pregnancy when Th2-type cytokines increased (e.g. SLE [55]). By contrast, in the post partum period, when the Th2-type cytokines decreases Th1 and Th17-type inflammatory and autoimmune disorders could get worse and Th2-type autoimmune disorders could improve. As the matter of fact, during pregnancy, SLE flares are associated with anti-phospholipids (aPL) antibodies, synergic changes of pregnancy on Th1 and Th2 cytokines, that interact with hormones such as estrogen and prolactin that amplify the inflammatory effect. From the clinical point of view, SLE activity at pregnancy onset, thrombocytopenia, lupus nephritis, arterial hypertension, aPL syndromes, preeclampsia is associated with lupus flares and fetal complications. The SLE flares during pregnancy make the difference between an uncomplicated pregnancy and pregnancy with maternal and fetal complications [55]. Pregnancy and the postpartum period have a profound effect not only on SLE, but also on autoimmune thyroid disease. Graves disease ameliorates during pregnancy, only to relapse postpartum, whereas postpartum thyroiditis is caused by destructive thyroiditis during the first few months after delivery [51]. Similarly, pregnant women with MS typically have a greatly reduced relapse rate, whereas very soon after the delivery, the disease activity returns, often even at a higher level than seen in the pre-pregnancy year. The reasons for the increased postpartum activity are not entirely clear, but factors such as the abrupt decrease in estrogen levels immediately after the delivery and the loss of the immunosuppressive state of pregnancy are likely of importance [54]. There is compelling evidence that estrogen, progesterone, and testosterone control autoimmune disorders. In particular these hormones control MS by influencing immune responses and by contributing to repair mechanisms in the nervous system. Similarly to many other autoimmune diseases, MS is more common among women than men, and its incidence among women is rising. There are also qualitative differences in the disease course between men and women, with male patients experiencing increased disease progression, brain atrophy and cognitive impairment.

Hormones, which seems to contribute to autoimmune disorders worsening or improvement by influencing T cell cytokine responses involved in the pathogenesis of these disorders, may offer important insights into autoimmune disease prevention and treatment (Table 1).

**Table 1:** Pregnancy influences autoimmune diseases progression

Autoimmune disease	Influence on pregnancy
Rheumatoid arthritis (RA)	Improved
Systemic lupus erythematosus (SLE)	Worse
Multiple sclerosis (MS)	Improved
Graves’ disease	Improved
Systemic sclerosis (SS)	Worse
Hashimoto thyroiditis	Improved

During pregnancy Th2-type autoimmune disease get worsen and Th1/Th17-type autoimmune disease improved

## Hormone-controlled T cell cytokine profile influences progression of autoimmune and inflammatory diseases

Women mount more vigorous antibody- and cell-mediated immune responses following either infection or vaccination than men. A large number of autoimmune diseases are more prevalent in women. Thus, it seems that sex influences susceptibility to autoimmune diseases (e.g. RA, for which the female-to-male ratio is 3:1 has been reported [56]). Many ideas mainly based on hormonal and genetic factors that influence the autoimmune systems of females and males differently, have been proposed to explain this predominance. These hypotheses have gained credence mostly because many of these diseases appear or fluctuate when there are hormonal changes such as in late adolescence and pregnancy. Differences in X chromosome characteristics between men and women with an autoimmune disease have led researchers to think that the genetic background of this group of diseases also relates to the genetic determinants of gender. Thus, the hormonal changes as well as the genetic factors, could explain why women are more prone to develop autoimmune diseases [57].

Female hormonal stimulation of immune responses could be responsible for the higher incidence of most autoimmune diseases in women than in men. Prior the discovery of Th17 cells, the first evidence of the role of hormonal changes on the autoimmune disorders progression derived from studies performed on Th1- and Th2- type cells. Indeed, it has been demonstrated that both androgens and estrogens are able to regulate the Th1/Th2 balance. Androgens were described as hormones which promote autoimmune diseases with a profile of Th1-type cytokines, such as RA, whereas estrogens were described as inducers of autoimmune diseases with a Th2-type cytokine profile, like SLE. Thus, Th1-type autoimmune diseases could be improved when decrease Th1-type cytokines, or when there is a rise in Th2-type cytokines (increased estrogens, as in pregnancy), whereas Th2-type autoimmune diseases could be improved when Th2-type cytokines are diminished (decreased estrogen, as in post-partum period) or when Th1-type response is stimulated [58].

This idea has gained credence because, in particular for RA, the peak incidence in women coincides with the time of menopause, when estrogen levels rapidly drop, connecting sex hormones to disease etiology [59]. In contrast, men have rather continuous levels of estrogen throughout their adult lives, and estrogen levels are lower in postmenopausal women than in men of corresponding age [60]. In a well-established experimental model of RA, the collagen-induced arthritis (CIA), it has repeatedly been shown that estrogen ameliorates disease development [61, 62]. In contrast to RA, estrogen aggravates systemic lupus erythematosus [63]. Estrogen is a potent immunomodulatory agent and can exert stimulatory as well as regulatory effects on the immune system, such as enhancing B cell antibody production, reducing B and T lymphopoiesis and inhibiting T cell-dependent inflammation [64, 65].

More recently with the discovery of Th17 cells, RA has been associated as MS, to Th17-mediated inflammatory diseases. Effects of estrogen on Th17 cells have mostly been studied in the context of the experimental MS, called EAE, where an estrogen is protective. Estrogen decreases production of IL-17 in EAE and inhibits Th17 differentiation and disease progression, dependent on estrogen receptor α (ERα) in T cells [66]. Furthermore, the regulatory role of estrogen has been demonstrated in CIA, where the estrogen metabolite 2-methoxyestradiol decreased IL-17 mRNA in arthritic joints [67], regulates the localization of Th17 cells, resulting in increased Th17 in lymph nodes but decreased Th17 in joints of CIA [56].

Progesterone is also able to decrease the Th17 cell activity [68] and could decrease the progression of IL-17-mediated autoimmune and inflammatory diseases. Progesterone is also a potent Th2-inducer [27] that could influence the progression of Th2 and Th1-mediated autoimmune disorders, by worsening Th2-type autoimmune disorders and improving Th1-mediated autoimmune and Th17/Th1-mediated inflammatory disorders. During pregnancy, the rise in Th2-type cytokines could be mostly due to progesterone, which is 10- to 50-fold higher at feto-maternal interface than in peripheral blood and which can inhibit the development and function of Th1 and macrophages cells [69], thus preventing the trophoblast alloantigen rejection.

**Fig. 1:**
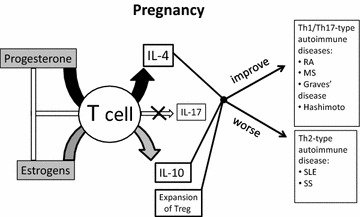
Hormone-controlled T cell responses influence the progression of autoimmune diseases: progesterone and estrogens inducing Th2-type response and decreasing Th1/Th17 response influence the improvement and the worsening of Th1/Th17-type autoimmune diseases, and Th2-type autoimmune diseases, respectively

Therefore, during pregnancy, hormonal changes which could play a critical role in determining the effector CD4+ T cell cytokine profile at fetomaternal interface, could explain why in some conditions pregnant women are more prone to develop Th2-type autoimmune diseases, whereas in pregnant women the Th1- and Th17-type autoimmune and inflammatory diseases are improved (Fig. 1).

## Conclusion

The immunology of pregnancy underlies the role of CD4+ T cell cytokine profile. Indeed, the mother must maintain tolerance of the fetal semi-allograft while not suppressing her own immune system and exposing herself and the fetus to infection. Non-specific factors, including probably not only hormonal changes [26, 27, 70], but also trophoblast key immunomodulatory molecules (e.g. HLA-G5, [10]) favoring a switch to a predominantly Th2-type cytokine profile, may play some part in the maintenance of transient tolerance to paternal antigens in pregnancy [23, 25, 26]. The generation of specific Th2 and Th17/Th2 cells and T reg cells, is key to the maintenance of pregnancy. These cells preferentially accumulate in the decidua but may also be present in the mother’s circulation and are thus capable of regulating coincidental autoimmune responses to remission or worsening of the related Th1-/Th17- or Th2-autoimmune diseases, respectively [51]. The postpartum exacerbation of some Th1/Th17 autoimmune disorders may reflect an imbalance in Th2-type cells and T reg cells, which is caused by the rapid fall in the numbers of these cells after delivery.

Autoimmune disorder development could also be influenced by the transplacental transfer of cells between mother and fetus, which occurred during pregnancy even years before the onset of the disorder. The fetal microchimerism is acquired by the mother during pregnancy and this fetal cells are maintained in the mother for decades. Similarly, there is also a maternal microchimerism acquired by an infant during pregnancy. In mouse models it is possible to study the bi-directional transfer of cells in pregnancy using fluorescent tags. Years ago, we showed that systemic sclerosis (SSc), an immunologically mediated disorder of the connective tissue, is characterized by a predominant activation of IL-4-producing Th2 cells, which may account for the clinical and serological similarities of SSc to chronic graft versus host disease (cGVHD), in which T cells from involved tissues produce Th2-type cytokines. We showed that offspring CD4+ T cells present in the blood and skin of women with SSc are able react with maternal MHC antigen and exhibit a Th2-type profile, supporting the possibility that cGVHD reaction attributable to long-term microchimerism plays a pathogenic role of SSc [71, 72]. The fetal microchimerism has been described in many other autoimmune diseases [73]. More recently, it has been demonstrated that maternal microchimerism could play a role in type 1 diabetes of children [74].

Therefore, pregnancy is able to influence the onset and progression of autoimmune and inflammatory diseases by influencing the T cell cytokine-mediated responses during the gestation period, the post-partum period, but also decades after the pregnancy period.

## Abbrevations

Th: T helper
APC: antigen presenting cell
IL: interleukin
HLA: human leukocyte antigen
TNF: tumor necrosis factor
IFN: interferon
URA: unexplained recurrent abortion
MS: multiple sclerosis
RA: rheumatoid arthritis
MHC: major histocompatibility complex
EAE: experimental allergic encephalomyelitis
TLR: toll like receptor
IDO: indoleamine 2,3-dioxygenase
ROS: reactive oxygen species
SLE: systemic lupus erythematosus
AHR: aryl hydrocarbon receptor
MG: myasthenia gravis
SS: Sjögren’s syndrome
CIA: collagen-induced arthritis
Erα: estrogen receptor α
SSc: systemic sclerosis
cGVHD: chronic graft versus host disease
